# A Note on Evaluation of Temporal Derivative of Hypersingular Integrals over Open Surface with Propagating Contour

**DOI:** 10.1007/s10659-014-9499-9

**Published:** 2014-09-24

**Authors:** Dawid Jaworski, Aleksandr Linkov, Liliana Rybarska-Rusinek

**Affiliations:** Rzeszow University of Technology, Powstancow Warszawy 12, 35-959 Rzeszow, Poland

**Keywords:** Propagating crack, Hypersingular integrals, Differentiation with respect to parameter, 30E20, 45E05, 74G70

## Abstract

The short note concerns with elasticity problems involving singular and hypersingular integrals over open surfaces, specifically cracks, with the contour propagating in time. Noting that near a smooth part of a propagating contour the state is asymptotically plane, we focus on 1D hypersingular integrals and employ complex variables. By using the theory of complex variable singular and hypersingular integrals, we show that the rule for evaluation of the temporal derivative is the same as that for proper integrals. Being applied to crack problems the rule implies that the temporal derivative may be evaluated by differentiation under the integral sign.

## Introduction

Using *singular* and *hypersingular* integrals and boundary integral equations (BIE) has proved to be a highly efficient means for solving problems of fluid and solid mechanics (see, e.g., [2–7, 9–11, 13, 15, 18]). The modern theories of hypersingular integrals and HBIE, both real and CV, are comprehensive when the boundary of the region of integration is fixed. However, there have arisen new computational problems involving propagating surfaces (see, e.g., [21]), which require formulae for temporal derivative of singular and hypersingular integrals. Such formulae are also needed for the sensitivity analysis applied to error estimation of the boundary element method [8]. In the case of non-singular integrals, the differentiation rules were given in [19]. They have been employed in [8] for obtaining the derivative of a singular integral over a surface of a 3D domain when points of the surface move in such a way that the initial domain stays globally unchanged (the changes in positions of the surface points occur in the tangential direction). This case is of prime significance when studying how the change of the position of a collocation point influences the value of a singular integral.

In the present paper, we are interested in another case, when the surface is open and the positions of its points behind a propagating contour do not change, while the contour and density change in time. We have come across such a problem when studying hydraulic fracturing widely used for stimulation of oil, gas and heat production (see, e.g., [1]). Then employing the temporal derivative of the hypersingular integral might notably facilitate numerical modeling of the fracture propagation in time. The main difficulty when obtaining the differentiation rule for this case is caused by the moving boundary rather than by the change of the density in time. Indeed, for a fixed contour, the common definition of the principal value or finite-part integral as the limit after exclusion a small *ε*-vicinity of a singular point, leads (under physically sound conditions on the smoothness of the surface, contour and density) to the conclusion that the differentiation may be performed under the integral sign. Consequently, for a moving crack front, we may fix a contour close to the front at the time instant considered and represent the integral as the sum of that with the fixed boundary and the integral over the thin strip between the fixed contour and the front. Thus the difficulty actually refers to differentiation of the integral over the thin strip. The latter integral involves the asymptotic behaviour of the density near the boundary. In applied problems, the *asymptotic behaviour is asymptotically plane*. This implies that to obtain the differentiation rule, it is reasonable to focus on 1D singular and hypersingular integrals of the plane potential and elasticity problems. Then the Cauchy-Riemann conditions for a harmonic function suggest using holomorphic functions having derivatives of an arbitrary order. This property is of key significance to connect the *limiting values* of the Cauchy type integral and Hadamard type integral, when the field point goes to the contour (surface) of integration, with the values of density and *direct* (principal, finite-part) *values* of Cauchy and Hadamard integrals. In real variables this beneficial property is reached by using the distribution theory (see, e.g., [16]).

Below we employ the advantages of the CV holomorphic functions in the CV variable and the theory of CV singular [17, 18] and hypersingular [13, 14] integrals to derive the needed rule for differentiation with respect to a parameter. Higher order hypersingular integrals are included into the rule because of their presence in efficient quadrature rules used in numerical solutions of hypersingular integrals (see, e.g., [13]). The evident extensions to singular and hypersingular integrals over an open surface with propagating contour are sketched in comments at the ends of Sects. 3 and 4.

## Starting Definitions

**Fig. 1:**
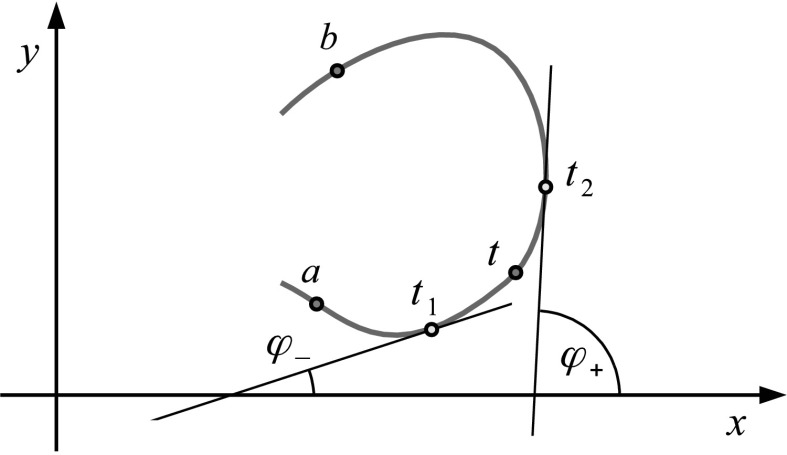
An open arc (*ab*), points *a*,*b*,*t*_1_,*t*,*t*_2_ and angles *φ*_−_,*φ*_+_

Consider an open curve (arc) in the complex plane *z*=*x*+*iy* ($i=\sqrt{-1}$) (Fig. 1). The equation of the arc is *τ*(*γ*)=*x*(*γ*)+*iy*(*γ*), where *γ* is a real parameter such that its value *γ*_*a*_ corresponds to start point *a*, while the value *γ*_*b*_ corresponds to end point *b*: *a*=*x*(*γ*_*a*_)+*iy*(*γ*_*a*_), *b*=*x*(*γ*_*b*_)+*iy*(*γ*_*b*_). The arc is smooth in the sense explained in [18]. Specifically,

- the functions *x*(*γ*) and *y*(*γ*) are continuous on the closed interval [*γ*_*a*_,*γ*_*b*_],
- they have continuous derivatives *x*′(*γ*) and *y*′(*γ*) on the open interval (*γ*_*a*_,*γ*_*b*_),
- the derivatives are not zero simultaneously, that is *x*′(*γ*)^2^+*y*′(*γ*)^2^>0 for *γ*∈(*γ*_*a*_,*γ*_*b*_),
- there are no branch-points on the arc what means that the simultaneous equalities *x*(*γ*_1_)=*x*(*γ*_2_) and *y*(*γ*_1_)=*y*(*γ*_2_) imply that *γ*_1_=*γ*_2_.

We accept these conditions and call such an arc a smooth arc.

In further discussion, the positions of start and end points may change depending on a real parameter *α*. (In applied problems the parameter is commonly the time.) Then *γ*_*a*_=*γ*_*a*_(*α*), *γ*_*b*_=*γ*_*b*_(*α*), *a*=*a*(*α*), *b*=*b*(*α*). The curve is smooth for each value of *α*.

Let a CV function *g*(*τ*) be prescribed at points of the arc (*a*,*b*). We assume it Holder continuous at (*a*,*b*), that is [18] there exist non-negative numbers *A* and *μ*≤1 such that |*g*(*τ*_1_)−*g*(*τ*_2_)| ≤*A*|*τ*_1_−*τ*_2_|^*μ*^ for any *τ*_1_,*τ*_2_∈(*a*,*b*).

Consider a hypersingular integral of order *k* over an arc [*a*,*b*]

1 $$ I_{k}(t)=\int_{a}^{b}\frac{g(\tau)}{(\tau-t)^{k}}d\tau. $$

We assume that the density *g*(*τ*) has (*k*−1)-th Holder continuous derivative with respect to *τ*. In the non-trivial case when *t*∈(*a*,*b*), the hypersingular integral is defined as [13]:

2 $$\begin{aligned} I_{k}(t)=\int_{a}^{b}\frac{g(\tau)}{(\tau-t)^{k}}d\tau =&\lim_{\varepsilon\rightarrow0} \Biggl[\int_{a}^{t_{1}}\frac{g(\tau)}{(\tau-t)^{k}}d\tau+\int_{t_{2}}^{b} \frac{g(\tau)}{(\tau-t)^{k}}d\tau \\ &{}-\sum_{m=1}^{k-1}\frac{(m-1)!}{(k-1)!}g^{(k-1-m)}(t) \frac{1-(-1)^{m}}{\varepsilon^{m}}e^{-im\varphi} \Biggr], \end{aligned}$$

where *t*_1_ and *t*_2_ are points on the arc located at the distance *ε* from the point *t* before and after this point, respectively, *φ* is the angle of the tangent at the point *t* with the *x*-axis (Fig. 1). In the case *k*=1, the sum on the r.h.s. of (2) is not present and the integral (1) is Cauchy principal value integral; in the case *k*=2, it is Hadamard finite-part integral. Equation (2) is obtained in the way, which provides the common Cauchy principal value integral in the case *k*=1. When *k*=2, it follows the line of introducing the Hadamard *finite-part* integral. Specifically, (i) a small *ε*-vicinity *L*_*ε*_ is excluded from the integration contour *L*, so that integration is performed over the part *L*−*L*_*ε*_, where the kernel is non-singular; (ii) successive integration by parts is used for the proper integral over *L*−*L*_*ε*_ until arriving at the integral with logarithmic kernel; (iii) only *finite parts* of the resulting out-of-integral terms are left (they do not depend on *ε*). This actually means subtraction of the terms going to infinity, when *ε*→0, from the integral over *L*−*L*_*ε*_, what is expressed by the sum in the brackets of (2). From the said it is obvious that (2) may be also written as:

3 $$\begin{aligned} I_{k}(t) =\int_{a}^{b}\frac{g(\tau)}{(\tau-t)^{k}}d\tau =&\frac{1}{(k-1)!}g^{(k-1)}(t)i\pi \\ &{}+\frac{1}{(k-1)!} \bigl[g^{(k-1)}(b)\ln(b-t) -g^{(k-1)}(a)\ln(a-t) \bigr] \\ &{}-\sum_{m=1}^{k-1}\frac{(m-1)!}{(k-1)!} \biggl[\frac{g^{(k-1-m)}(b)}{(b-t)^{j}} -\frac{g^{(k-1-m)}(a)}{(a-t)^{j}}\biggr] \\ &{}-\frac{1}{(k-1)!}\int_{a}^{b}g^{(k)}(\tau)\ln(\tau-t)d\tau. \end{aligned}$$

Equation (3) shows that the integral *I*_*k*_(*t*) is a Holder continuous function of *t* on (*a*,*b*) (for a singular integral (*k*=1), the sum on the r.h.s. of (3) is omitted).

Consider a density *g*(*α*,*τ*) depending on the same parameter *α* as the limits of integration. The integral becomes a function of *α*:

4 $$ I_{k}(\alpha,t)=\int _{a(\alpha)}^{b(\alpha)}\frac{g(\alpha,\tau)}{(\tau-t)^{k}}d\tau. $$

Assume that the density and its derivatives up to the order *k*−1 with respect to *τ* have Holder continuous derivative with respect to *α* for any *τ*∈(*a*,*b*). In this case, from (3) it follows that the integral has Holder continuous partial derivative with respect to *α*, which may be evaluated by direct differentiation of the r.h.s. of (3) with respect to *α*. In the case, when the limits do not depend on the parameter, the inspection of the result of differentiation leads to the conclusion that partial differentiation with respect to the parameter may be performed under the integral sign.

## Formula for Differentiation of a Hypersingular Integral with Respect to a Parameter

Denote *J*_*g*_(*α*,*τ*) an antiderivative of the integrand $\frac{g(\alpha,\tau)}{(\tau -t)^{k}}$ in (4). This means that

5 $$ \frac{\partial J_{g}(\alpha,c)}{\partial c}=\frac{g(\alpha ,c)}{(c-t)^{k}}. $$

Differentiating (5) with respect to *α* yields:

6 $$ \frac{\partial}{\partial c} \biggl( \frac{\partial J_{g}(\alpha ,c)}{\partial \alpha} \biggr) =\frac{\frac{\partial g(\alpha,c)}{\partial\alpha }}{(c-t)^{k}}. $$

Herein, we have changed the order of differentiation on the l.h.s. what is justified under the conditions accepted.

The following derivation shows that commutation of operations of integration and differentiation with respect to a parameter is applicable. It is based on the extended Newton-Leibnitz (N-L) formula proved in [13] under the explained definition of the hypersingular integral. As the integrand of *I*_*k*_(*α*,*τ*) has the antiderivative *J*_*g*_(*α*,*τ*), the extended N-L formula, applied to (4), reads:

7 $$ \int_{a(\alpha)}^{b(\alpha)}\frac{g(\alpha,\tau)}{(\tau -t)^{k}}d\tau =J_{g}(\alpha,b)-J_{g}(\alpha,a)+\frac{i\pi}{k!}g_{t}^{(k-1)}( \alpha,t), $$

where $g_{t}^{(k-1)}(\alpha,t)$ is the (*k*−1)-th partial derivative of *g*(*α*,*t*) with respect to the argument *t*.

Similarly, when using the antiderivative $\frac{\partial J_{g}(\alpha ,c)}{\partial\alpha}$, the extended N-L formula reads:

8 $$ \int_{a(\alpha)}^{b(\alpha)}\frac{\frac{\partial g(\alpha,\tau )}{\partial\alpha}}{(\tau-t)^{k}}d\tau= \frac{\partial J_{g}}{\partial \alpha}(\alpha,b)-\frac{\partial J_{g}}{\partial\alpha}(\alpha ,a)+\frac{i\pi}{k!} \frac{\partial g_{t}^{(k-1)}}{\partial\alpha}(\alpha,t). $$

Differentiation of the both parts of (7) with respect to *α* gives:

9 $$\begin{aligned} \frac{\partial}{\partial\alpha}\int_{a(\alpha)}^{b(\alpha)}\frac {g(\alpha,\tau)}{(\tau-t)^{k}}d\tau =&\frac{\partial J_{g}}{\partial\alpha }(\alpha,b) -\frac{\partial J_{g}}{\partial\alpha}(\alpha,a) +\frac{i\pi}{k!}\frac{\partial g_{t}^{(k-1)}}{\partial\alpha}(\alpha,t) \\ &{}+\frac{\partial J_{g}}{\partial b}\frac{db}{d\alpha}-\frac{\partial J_{g}}{\partial a}\frac{da}{d\alpha}. \end{aligned}$$

In view of (8) and (5), equation (9) becomes:

10 $$\begin{aligned} &\frac{\partial}{\partial\alpha} \int_{a(\alpha)}^{b(\alpha)}\frac{g(\alpha,\tau)}{(\tau-t)^{k}}d\tau \\ &\quad =\int_{a(\alpha)}^{b(\alpha)} \frac{\partial g(\alpha,\tau)}{\partial\alpha} \frac{d\tau}{(\tau-t)^{k}} +\frac{g(\alpha,b)}{(b-t)^{k}}\frac{db}{d\alpha} -\frac{g(\alpha,a)}{(a-t)^{k}}\frac{da}{d\alpha}. \end{aligned}$$

We have proved the theorem expressing the rule of differentiation of a hypersingular integral with respect to a parameter.

### Theorem

*For a smooth* *arc*(*a*,*b*) *with* *a*(*α*) *and* *b*(*α*) *being Holder continuous in a parameter* *α* *and for a density* *g*(*α*,*τ*) *having* (*k*−1)-*th Holder continuous derivative with respect to* *τ* *and Holder continuous derivative with respect to* *α*, *the derivative of a hypersingular integral* *I*_*k*_(*α*,*t*) *with respect to the parameter* *α* *has the form* (10) *reproducing the common rule for proper integrals*.

### Remark 1

Similar rule holds for 2D singular and hypersingular integrals over an open surface with a propagating front.

## Extension to Densities with Derivatives Having Power-Type Singularity at Arc Tips

In applied problems concerning with cracks, *k*=2 and *α* has the meaning of the time. Commonly, the integral on the l.h.s. of (7) is proportional to the net-pressure on crack surfaces, the density *g*(*α*,*τ*) is the fracture opening and the derivatives *db*/*dα* and *da*/*dα* express the speeds, with which the fracture front propagates. According to (10), the influence of the speeds on the rate of the pressure change strongly depends on the values *g*(*α*,*a*) and *g*(*α*,*b*) of the opening at the points of the front *a* and *b*. Usually, near a point *c* of the front, the opening tends to zero as (*c*−*τ*)^*γ*^, where *Re*(*γ*)>0. In particular, in fracture mechanics, commonly *γ*=0.5 (see, e.g., [20]); in problems of hydraulic fracture, propagating in the viscosity dominated regime, *γ*=2/3 (see, e.g., [22]); for the leak-off dominated regime, *γ*=5/8 (see, e.g., [12]). Hence, we need to extend the theorem to the case when near an edge point *c* (*c*=*a* or *c*=*b*) the density is of the form *g*(*α*,*τ*)=(*c*−*τ*)^*γ*^ *g*_*γ*_(*α*,*τ*), where *Re*(*γ*)>0 and the function *g*_*γ*_(*α*,*τ*) meets the conditions of the theorem. Note that *g*(*α*,*c*)=0.

We may represent the integral (4) as the sum of three integrals

11 $$ \int _{a(\alpha)}^{b(\alpha)}\frac{g(\alpha,\tau)}{(\tau-t)^{k}}d\tau =\int _{a_{1}(\alpha)}^{b_{1}(\alpha)} \frac{g(\alpha,\tau)}{(\tau-t)^{k}}d\tau+\int _{a(\alpha)}^{a_{1}(\alpha)}\frac{g(\alpha ,\tau)}{(\tau-t)^{k}}d\tau+\int _{b_{1}(\alpha)}^{b(\alpha)} \frac{g(\alpha,\tau)}{(\tau-t)^{k}}d\tau, $$

where *a*_1_(*α*) is an arbitrary point between *a*(*α*) and *t*, while *b*_1_(*α*) is an arbitrary point between *t* and *b*(*α*). The first of them does not contain the edges as points of integration; hence the general theory and the proved theorem are applicable to it. Two remaining integrals are usual proper integrals because the point *t* does not belong to their intervals of integration; their partial derivatives with respect to *α* may be evaluated in a common way because, under the assumptions, the partial derivative *∂g*(*c*,*τ*)/*∂c* is integrable. This implies the extension of the theorem.

### Extended Theorem

*For a density having representation* *g*(*α*,*τ*)=(*c*−*τ*)^*γ*^ *g*_*γ*_(*α*,*τ*) *near start* (*c*=*a*) *and end* (*c*=*b*) *points*, *the theorem holds for points* *t* *within an open arc* (*ab*).

Since *g*(*α*,*c*)=0, the equation (10) becomes:

12 $$ \frac{\partial}{\partial\alpha} \int_{a(\alpha)}^{b(\alpha)} \frac{g(\alpha,\tau)}{(\tau-t)^{k}}d\tau=\int_{a(\alpha )}^{b(\alpha)} \frac{\partial g(\alpha,\tau)}{\partial\alpha} \frac{d\tau}{(\tau -t)^{k}}. $$

The differentiation formula (12) means that it is possible to differentiate under the integral sign. This result is of special significance in problems of fracture mechanics; in these problems, *k*=2 and 0<*Re*(*γ*)<1.

Equation (12) may be easily checked by direct evaluation of its left and right hand sides in cases of the Cauchy principal value (*k*=1) and Hadamard finite part (*k*=2) integrals when the density is of the form

$$g(\alpha,\tau)=P_{n}(\alpha,\tau)\sqrt{\bigl[\tau-a(\alpha) \bigr] \bigl[b(\alpha)-\tau\bigr]}, $$

where $P_{n}(\alpha,\tau)=\sum_{j=0}^{n}d_{j}(\alpha)\tau^{j} $ is a polynomial of degree *n* with coefficients depending on the parameter *α*. Then the Cauchy integrals on the both parts of (12) are evaluated analytically by using equations given in Sect. 110 of [18]; analogous formulae for the Hadamard integrals are promptly obtained by integration by parts.

It appears (see Appendix) that when the distance *d* between the point *t* and a tip *c* goes to zero, the integral (12) behaves as *O*(*d* ^−*k*+*γ*^), when *γ*≠1/2; it is non-singular, when *γ*=1/2.

### Remark 2

In applied problems for 2D surfaces, the asymptotic behaviour of the crack opening near a smooth part of a contour is the same as in a plane-strain problem. Consequently, integration under the integral sign is possible in these problems, as well.

## Summary

The results of the paper are summarized as follows. Assume that the density, the integration domain and its boundary are sufficiently smooth functions of the spatial coordinate(s) and time. (For 1D problems, the exact meaning of smoothness is explained in Sect. 2.) Then

- the temporal derivative of singular and hypersingular integrals may be evaluated by the common rule for proper integrals;
- it is possible to evaluate the temporal derivative under the integral sign when either the boundary is fixed, or the density is zero on the moving boundary; in these cases, the singular and hypersingular boundary integral equations keep their from for temporal derivatives of physical quantities;
- near a smooth part of a propagating boundary, the temporal derivative of a hypersingular integral of order *k* asymptotically behaves as *O*(*d* ^−*k*+*γ*^), when the density asymptotically behaves as *O*(*d* ^*γ*^) with 0<*γ*<1, *γ*≠1/2 and *d* being the distance from the boundary. The temporal derivative is non-singular near a smooth part of the boundary if *γ*=1/2.

## Appendix: Analysis of Asymptotic Behavior of the Derivative

Assume that the density *g*(*α*,*τ*) has the form discussed in Sect. 4: *g*(*α*,*τ*)=(*τ*−*c*)^*γ*^ *g*_*γ*_(*α*,*τ*), where *c* is a crack tip, *Re*(*γ*)>0 and *g*_*γ*_(*α*,*τ*) satisfies the conditions of the theorem. For certainty, when considering asymptotics of the derivative of a hypersingular integral, we take *c*=*a*, *Re*(*γ*)<1, *k*≥2 (in view of (3), the case *k*=1 is covered by the theory of [17]). Then the equation (12) may be written as

13 $$\begin{aligned} &\frac{\partial}{\partial\alpha} \int_{a(\alpha)}^{b(\alpha)} \frac{g(\alpha,\tau)}{(\tau-t)^{k}}d\tau \\ &\quad =-\gamma\frac{da}{d\alpha}\int_{a(\alpha)}^{b(\alpha)} \frac{g_{\gamma }(\alpha,\tau)d\tau}{(\tau-a)^{\beta}(\tau-t)^{k}} +\int_{a(\alpha )}^{b(\alpha)} \frac{(\tau-a)\frac{\partial g_{\gamma}}{\partial\alpha}d\tau}{(\tau-a)^{\beta}(\tau-t)^{k}}, \end{aligned}$$

where *β*=1−*γ* and the function $(\tau-a)\frac{\partial g_{\gamma} }{\partial\alpha}$ meet the conditions of the theorem. Note that 0<*Re*(*β*)<1 because 0<*Re*(*γ*)<1. From (13), it is clear that the asymptotics of the derivative is defined by the first integral on the r.h.s. We may write *g*_*γ*_(*α*,*τ*) as *g*_*γ*_(*α*,*τ*)=[*g*_*γ*_(*α*,*τ*)−*g*_*γ*_(*α*,*t*)]+*g*_*γ*_(*α*,*t*) and take into account that the difference is *g*_*γ*_(*α*,*τ*)−*g*_*γ*_(*α*,*t*) equals to zero at *τ*=*t* and has Holder continuous derivative at this point. Therefore, when studying the asymptotic behaviour of the derivative, it is sufficient to distinguish the asymptotics of the integral:

14 $$ \varPhi_{k}(a,t)=\frac{1}{2\pi i}\int_{a}^{b}\frac{d\tau}{(\tau-a)^{\beta}(\tau-t)^{k}}. $$

From the general theory [13, 14] it follows:

15 $$ \varPhi_{k}(a,t)=\frac{1}{(k-1)!}\frac{d^{k-1}}{dt^{k-1}} \frac{1}{2\pi i}\int_{a}^{b}\frac{d\tau}{(\tau-a)^{\beta}(\tau-t)}. $$

The integral on the r.h.s of (14) may be evaluated by using Muskhelishvili’s result [17]. Then (15) takes the form:

16 $$ \varPhi_{k}(a,t)=\frac{1}{(k-1)!}\frac{d^{k-1}}{dt^{k-1}} \Biggl( \frac{\cot \beta\pi}{2i}(t-a)^{-\beta}+\sum_{j=0}^{\infty}A_{j}(a) (t-a)^{j} \Biggr), $$

where *A*_*j*_(*a*) are certain coefficients (*j*=0,…). Note that (16) shows that in the case *β*=1/2 corresponding to *γ*=1/2, the function *Φ*_*k*_(*a*,*t*) is an analytical function; as such, it has no singularity at the tip *a*. Hence, it remains to study the case when *β*≠1/2 (*γ*≠1/2). In this case, after differentiation in the r.h.s of (16) we obtain:

17 $$\begin{aligned} \varPhi_{k}(a,t) =&\frac{1}{(k-1)!}\frac{(-1)^{k-1}\cot\beta\pi}{2i}\beta(\beta+1)\cdots(\beta+k-2) (t-a)^{-\beta-k+1} \\ &{}+\sum_{j=k-1}^{\infty}{n \choose{k-1}} A_{j}(a) (t-a)^{j-k+1}. \end{aligned}$$

Recalling that *β*=1−*γ*, we see from (17) that, when *γ*≠1/2, the asymptotic behaviour of the derivative (13) is *O*((*t*−*a*)^−*k*+*γ*^).
